# Inhibiting effect of miR-29 on proliferation and migration of uterine leiomyoma via the STAT3 signaling pathway

**DOI:** 10.18632/aging.203873

**Published:** 2022-02-02

**Authors:** Dai Huang, Hongyuan Xue, Weihua Shao, Xiaoxi Wang, Hongjuan Liao, Yuquan Ye

**Affiliations:** 1 Department of Medical Imaging and Department of Ultrasound, Hebei Medical University and Hebei General Hospital, Shijiazhuang 050051, Hebei, China; 2 Department of Ultrasound, Hebei General Hospital, Shijiazhuang 050051, Hebei, China; 3 Department of Second Division of Geriatrics, Hebei General Hospital, Shijiazhuang 050051, Hebei, China; 4 Department of Medical Examination Center, Hebei General Hospital, Shijiazhuang 050051, Hebei, China; 5 Department of Vascular Surgery, The Second Hospital of Hebei Medical University, Shijiazhuang 050000, Hebei, China; 6 Ultrasound Department, Hebei Medical University and Hebei General Hospital, Shijiazhuang 050051, Hebei, China

**Keywords:** miR-29, STAT3, uterine leiomyoma, proliferation, migration

## Abstract

Aim: Uterine leiomyoma is the most common benign tumor of female genitalia, and the incidence is rising gradually. This study explores the mechanism of miR-29 and STAT3 signaling pathways on uterine leiomyoma.

Methods: GSE64763 and GSE5244 datasets were downloaded. Enrichment analyses were performed in GSE64763. PPI network was constructed, and the significant module was identified. Uterine leiomyoma cell lines were divided into NC, miR-29 mimic, anti-NC, and miR-29 inhibitor groups. Plate clone formation and Transwell assays detected the proliferation, invasion, and migration of cells. The expression levels of STAT3, proliferation, EMT, invasion-associated proteins were determined by Western blotting.

Results: Differently expressed genes were mainly enriched in positive regulation of cell migration and gene expression, cell proliferation. Through GSEA, JAK-STAT is a significantly correlated enrichment pathway. A Venn diagram was drawn to identify the common miRNA (miR-29-3p). miR-29 inhibitors promoted protein expression of STAT-3, Cyclin D1, and c-Myc compared with the anti-NC control (P < 0.01), and miR-29 inhibitors promoted cell proliferation in uterine leiomyoma cells (P < 0.05). Furthermore, miR-29 inhibitors promoted the protein expression of MMP-2 and MMP-9 (P < 0.01), and EMT promoting proteins N-cadherin, snail, vimentin, and Transwell assay showed that miR-29 inhibitors promoted cell migration in uterine leiomyoma (P < 0.01).

Conclusions: High expression of miR-29 could inhibit cell proliferation, invasion, and metastasis in uterine leiomyoma, which might be related to the inhibition of the STAT3 signaling pathway, and could provide a novel target for the treatment of uterine leiomyoma.

## INTRODUCTION

Uterine leiomyoma, composed of smooth muscle and connective tissue, is the most common benign tumor in women (aged 30 to 50 years) [1]. In China, the incidence rate of uterine fibroids is the latest national statistics. The incidence rate of uterine fibroids is 30% in women aged 30 to 50 years. Clinically, it is ignored because the number of myomas is small, the volume is small, and there is no irregular menstruation or other symptoms. Hysteromyoma is common in women aged 30 ~ 50, rare under 30, and very rare under 20. The incidence of hysteromyoma is the highest in women aged 40 ~ 50, accounting for about 51.2 %~ 60.9% [2]. Patients with uterine leiomyoma might have symptoms of abnormal uterine bleeding, masses in the lower abdomen, and others [3]. The treatment of hysteromyoma is comprehensively considered according to the patient's age, fertility requirements, symptoms, location, and size, and surgical management, hysteroscopic surgery, and drug therapy are generally used. In severe cases, hysterectomy is needed, which will affect the reproductive health of women and increase the economic burden. The current medical management alleviates the clinical symptoms of patients with severe hysteromyoma. Surgical treatment could be the cure, but not suitable for all patients. Early detection, early diagnosis, and early treatment are critical [4]. However, the pathogenesis and etiology of uterine leiomyoma are still unclear. The disease might be related to genetic factors, chromosomal abnormalities, and other factors [5]. Therefore, it is vital to study the molecular mechanism of uterine leiomyoma.

Bioinformatics is an interdisciplinary subject that uses computer science as a tool to store, retrieve, analyze and interpret biological data [6, 7]. In recent years, with the rapid development of high-throughput sequencing technology, researchers can quickly obtain detailed analysis of the whole transcriptome [8]. With the increasing research on the gene expression profile of uterine leiomyoma, further research on uterine leiomyoma could be carried out through bioinformatics methods to expand the understanding of the pathological mechanism of uterine leiomyoma [9].

STAT3 forms part of the JAK-STAT signaling cascade and bases many cytokine receptor signaling mechanisms. Receptor-associated kinases phosphorylate STAT3 and form homozygous or heterodimer, which is transferred to the nucleus as a transcriptional activator [10]. This protein mediates the response of multiple genes to cellular stimuli. It, therefore, plays a crucial role in many cellular processes, including cell growth and apoptosis. MiRNAs are a class of small non-coding RNAs with a length of 19-24 nucleotides. MiRNAs play a biological role by negatively regulating gene expression at the post-transcriptional level [11, 12]. Studies have shown that miR-29 might inhibit the proliferation and regulate cell differentiation of tumor cells by targeting insulin-like growth factor-1 [13]. However, it is not clear whether miR-29 could target STAT3 to regulate the biological characteristics of cell proliferation and invasion in uterine leiomyoma.

Therefore, this study intends to use bioinformatics technology to excavate the core genes between uterine leiomyoma and normal uterine tissues and conduct enrichment analysis and pathway analysis. The significant role of miR-29 and STAT3 in uterine leiomyoma was verified using public data sets. Basic cell experiments were used to explore the potential regulatory mechanism of miR-29b on STAT3 in uterine leiomyoma to provide basic research for clinical treatment.

## MATERIALS AND METHODS

### Data acquisition

The gene expression datasets of uterine leiomyoma, GSE64763 [GPL571 platform (Affymetrix Human Genome U133A 2.0Array)], were downloaded in the GEO (Gene Expression Omnibus, https://www.ncbi.nlm.nih.gov/gds/) database. GSE5244 [GPL3879 platform] was about the miRNA sequencing dataset of uterine leiomyoma. Limma package under R language was used to analyze differentially expressed genes (|logFC|>1, P-value<0.05), and principal component analysis (PCA) and cluster analysis were performed simultaneously. The GGplot2 software package was used to construct the visual volcano map of the GSE64763 and GSE5244 datasets under the R language. The PHEATMAP package was used to draw the cluster analysis heatmap of differentially expressed genes (DEGs).

### Functional enrichment analysis

GO (Gene Ontology) enrichment analysis, and Kyoto Encyclopedia of Genes and Genomes (KEGG) enrichment analysis was performed on the DEGs in the GSE64763 dataset. By DAVID tool (https://david.ncifcrf.gov/), differentially expressed genes in the level of biological processes (BP) were analyzed by integrating the GO term, and the BP network was created. GGplot2 and GOplot packages were used to perform the GO and KEGG pathway enrichment analysis of DEGs in the R language environment.

### Gene Set Enrichment Analysis (GSEA)

Gene Set Enrichment Analysis (GSEA, http://www.gsea-msigdb.org/) is an enrichment analysis method based on Gene sets. One or more functional gene sets from MSIgDB were selected in this analysis. Then the sequencing was conducted based on the correlation degree between gene expression data and phenotype. Furthermore, it was determined whether the genes in each gene set were enriched in the upper or lower part of the gene list after sequencing of phenotypic correlation to determine the effect of synergistic changes of genes in this gene set phenotypic changes. GSEA enrichment analysis was performed on all genes using GSEA, so a GSEA enrichment analysis pathway map could be drawn.

### Protein-protein interaction (PPI) network and the significant module

The Search Tool for the Retrieval of Interacting Genes (STRING) (http://string-db.org) can convert DEGs into expressed proteins and construct a PPI network. We got a PPI network of common DEGs through STRING and visualized it by Cytoscape (version 3.7.2). Molecular Complex Detection tool (MCODE) (version 1.6.1), an open plug-in of Cytoscape, was performed to identify significant modules from the PPI network. The criteria were that the maximum depth=100, MCODE scores >5, cut-off=2, k-score=2, and node score cut-off=0.2.

### Predicting miRNA of target gene

TargetScan, TarBase, and mirDIP predicted the miRNA, and the target genes were predicted with the VennDiagram package. The binding sites between mRNA and miRNA were plotted according to the gene prediction results.

### Tissue isolation of primary uterine leiomyoma endometrial epithelial cells

Primary uterine leiomyoma endometrial epithelial cells were isolated from a few uterine leiomyomas, and cultured using the digestion and tissue adhesion methods previously described [14]. After chopping and homogenizing the tissues, the cells were digested in 1mg/mL collagenase IV for 3-5 h. The cells were successfully filtered through 100 μ m and 40μ m cell filters and washed with PBS containing 1% antibiotic solution 3 times. The cells were centrifuged at 460 g for 5 min, and the sediment was collected and suspended in Dulbecco modified Eagle medium (DMEM).

### HTP6136 cell culture and transfection

HTP6136 cells (Human uterine leiomyoma cell line) Cells purchased from Procell Life Science and Technology Co., Ltd. should be cultured in a cell incubator. Most cells were cultured at 37C, 5% 002, and 100% humidity. There were very few cell culture conditions that were inconsistent. The cells were cultured in a culture flask or dish. An appropriate amount of complete medium for the cells indicated on the instructions was added. Then the cells were cultured to a density above 80%, which can be sub-cultured and then digested with 0.125% trypsin and collected by centrifugation. The experiments were carried on when the cells were in the log growth phase. The miR-29 mimic or miR-29 NC, miR-29 inhibitor were co-transfected into HTP6136 cells with lipofectamine 3000. The cells were harvested after 48 h of culture. Mimic-NC: UUG UAC UAC ACA AAA GUA CUG GUA CUU UUG UGU AGU ACA AUU; miR-29-3p mimics: UAG CAC CAU CUG AAA UCG GUU A UA ACC GAU UUC AGA UGG UGC UA; inhibitor-NC: UCU ACU CUU UCU AGG AGG UUG UGA; miR-29-3p inhibitor: UAA CCG AUU UCA GAU GGU GCU A.

### Monoclonal proliferation

HTP6136 cells in the logarithmic growth phase were digested with trypsin, then resuspended in a complete culture medium and counted. The cells were seeded in the six-well plate at 400 cells/well density until the number of cells in most single clones was more than 50. The medium was changed every three days, and the cell status was observed. After cloning, the cells were washed with phosphate-buffered saline (PBS) and fixed with 4% paraformaldehyde for 30 minutes. After washing with PBS, added 0.1% crystal violet dye to each well, dyed the cells for 10-20 minutes, washed with PBS several times, dried, and took photos with the digital camera.

### Western blot

The cells cultured in the 6-well plate were added to ristocetin-induced platelet agglutination (RIPA) for lysis. The supernatant was centrifuged after the lysis was completed. The BCA kit was used to detect the protein concentration of each group of cells. The target protein sample was heat-denatured before being put into a 12 percent sodium dodecyl sulfate-polyacrylamide gel electrophoresis (SDS-PAGE) gel for electrophoresis separation and transfer to PVDF membranes. After the transfer was completed, the membrane was immersed in 5% skimmed milk powder and sealed for 2 hours. The PVDF membrane was incubated with primary antibodies including MMP-2(ab92536, Abcam, 1:1000 dilution), MMP-9(ab58803, Abcam, 1:1000dilution), STAT3(ab68153, Abcam, 1:800dilution), cyclin D1(ab16663, Abcam, 1:1000dilution), c-Myc (ab32072, Abcam, 1:1000dilution), β-actin(66009-1-Ig, protein each, 1:5000)which was used as a control at 4° C overnight. Shaked and washed 3 times in TBS solution and incubated with secondary antibodies at 24° C for 1h. Immune complexes were detected by the Western bands and were analyzed by ImageJ software.

### Transwell

The Matrigel diluted with the serum-free medium was spread on a polycarbonate microporous membrane with a pore size of 8 μm in the Transwell chamber and fully fused for 2 h. Collected the previously grouped cells and culture them were serum-starved for 24 h. Washed the treated cells twice with PBS, trypsinization, and centrifugation, resuspended the cells in an FBS-free medium and adjusted the number of cells to 5×10^3^/mL. Put Transwell chamber into a 24-well plate. Added 500 μl of the serum-containing medium in the lower chamber of the Transwell chamber, and 200μl of cell suspension in the upper chamber, and incubated for 24 h in the cell incubator. Cells were allowed to invade through the membrane for 24 hr in a serum-free growth medium in the incubate at 37° C, toward the lower chamber growth. Took out the chamber and discarded the upper chamber liquid. After washing with PBS, fixed the cells with formaldehyde. They have wiped off the upper cells of the chamber with a cotton swab, fixed with stained with 20% methanol/0.1% crystal violet for 10-15 minutes, washed with tap water and air dry, and counted under a microscope. Three independent experiments were performed, each in triplicate.

### Statistics

DEseq2 and ggpubr packages in the R (v3.6.1) software were used for statistical analysis. Differential analysis was performed using the Wald test. A rank-sum test was used to compare cytokines between two groups. P < 0.05 was considered statistically significant.

### Data availability

The data used to support the findings of this study are included in the article.

### Ethics approval

The animal use protocol for this study has been reviewed and approved by the Laboratory Human Ethical Committee, Hebei General Hospital (NO. 202199).

## RESULTS

### Screening of DEGs

**Figure 1:**
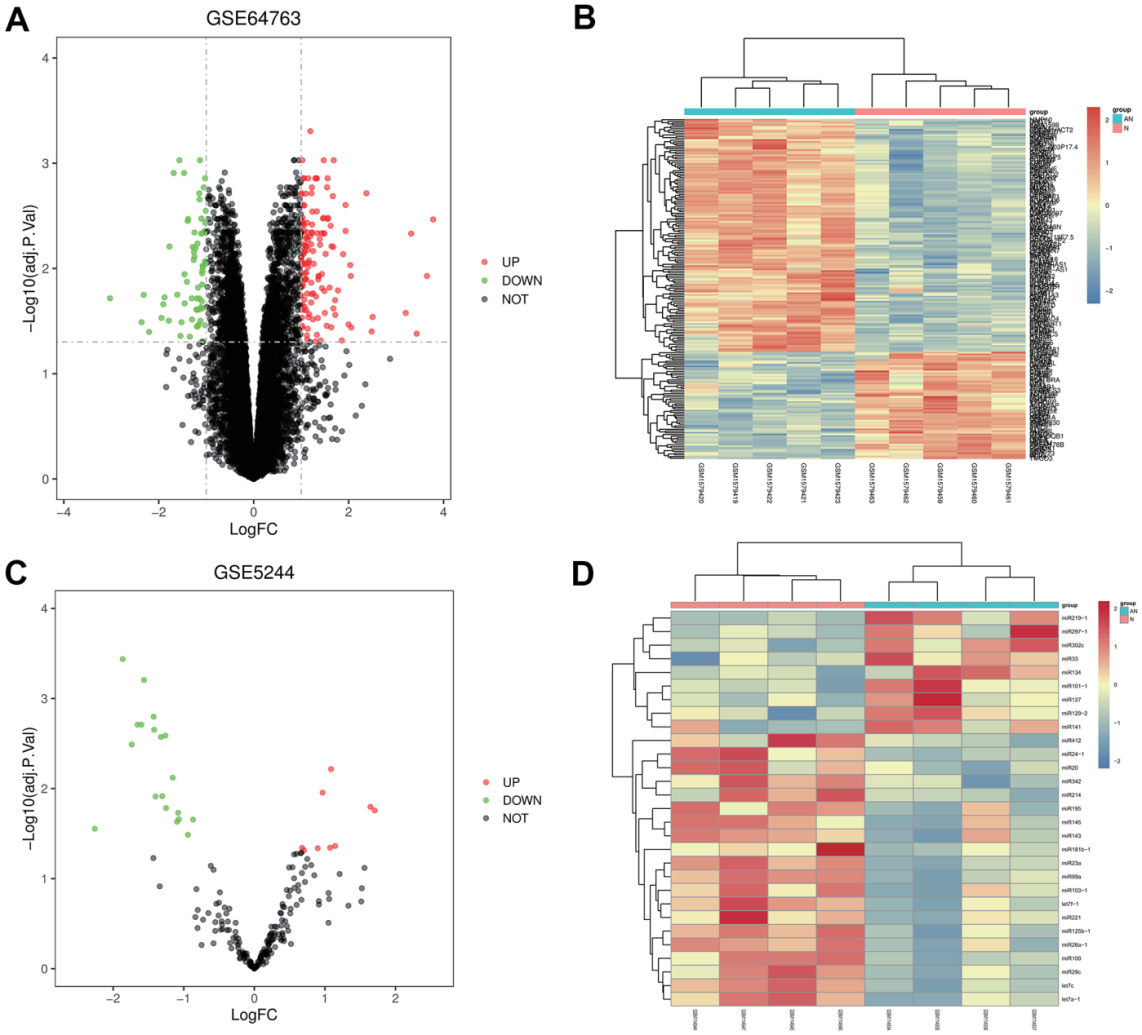
**Screening of differentially expressed genes (DEGs).** (**A**, **B**) Volcanic map and heatmap of DEGs in GSE64763. (**C**, **D**) Volcanic map and heatmap in GSE5244.

GSE64763 dataset, related to uterine leiomyoma, and GSE5244, one miRNA dataset, were downloaded from the GEO database. According to criteria of p-value<0.05 and |logFC|<1, DEGs were screened in GSE64763. A total of 204 DEGs were found in uterine leiomyoma, among which 139 were up-regulated, and 65 were down-regulated. The ggplot2 package was used to construct the visual volcano map of GSE64763 in R software (Figure 1A). The cluster analysis heatmap of DEGs was drawn via the pheatmap package (Figure 1B). The same method was used to construct a visual volcano map (Figure 1C) and cluster heatmap (Figure 1D) for the GSE5244.

### Enrichment analysis for the DEGs in GSE64763

**Figure 2:**
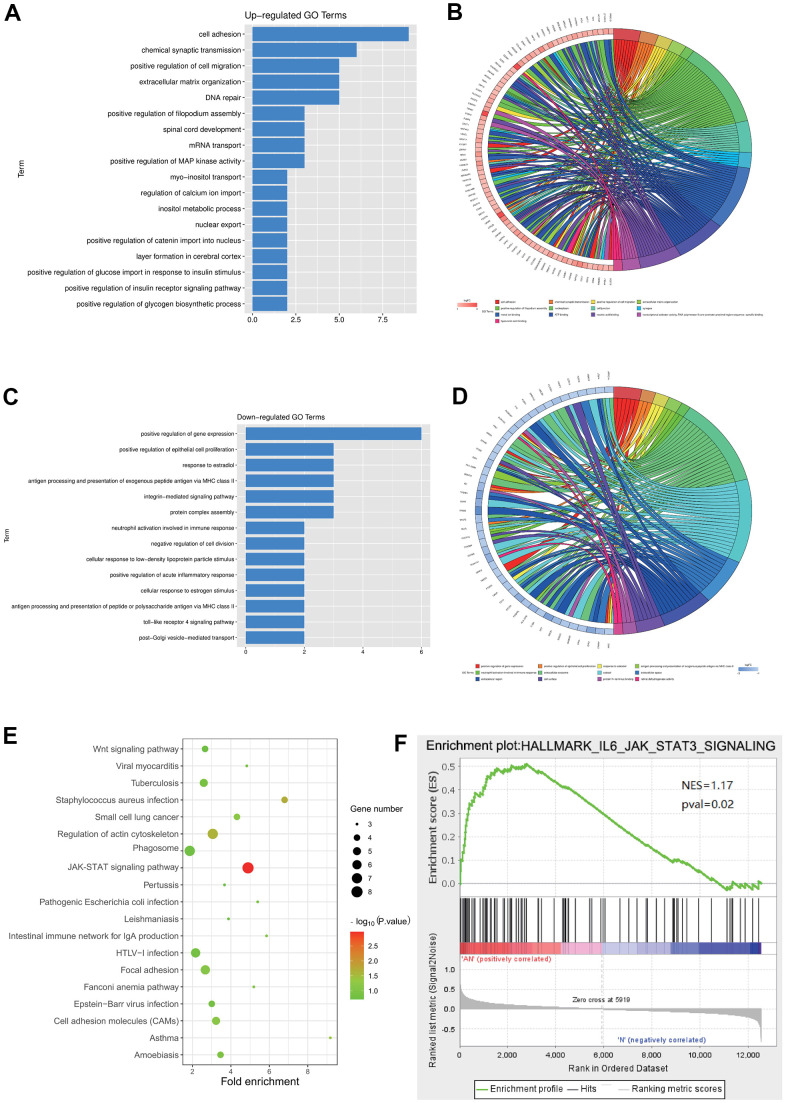
**Enrichment analysis for the DEGs in GSE64763.** (**A**, **B**) Enrichment maps of the up-regulated DEGs. (**C**, **D**) GO enrichment of the down-regulated genes was enriched in positive epithelial cell proliferation regulation. (**E**) KEGG showed that the main enriched pathways included JAK-STAT signaling pathway. (**F**) GSEA analysis manifested the JAK-STAT3 signaling pathway.

Go and KEGG enrichment analyses of the DEGs in the GSE64763 dataset were performed. R language was used to draw enrichment maps of the up-regulated DEGs, which were mainly enriched in the cell adhesion, chemical synaptic transmission, positive regulation of cell migration, positive regulation of filopodium assembly, spinal core development, mRNA transport, positive regulation of MAP kinase activity, and inositol metabolic process (Figure 2A, 2B). GO enrichment of the down-regulated genes were enriched in positive regulation of epithelial cell proliferation, response to estradiol, antigen processing and presentation of exogenous peptide antigen via major histocompatibility complex (MHC) class II, integrin-mediated signaling pathway, negative regulation of cell division, and positive regulation of acute inflammatory response (Figure 2C, 2D). KEGG showed that the main enriched pathways included Wnt signaling pathway, JAK-STAT signaling pathway, and focal adhesion (Figure 2E). In addition, through GSEA analysis, it was concluded that the JAK-STAT3 signaling pathway was a significantly enriched pathway for uterine leiomyoma (Figure 2F).

### STAT3 as the hub genes for uterine leiomyoma

**Figure 3:**
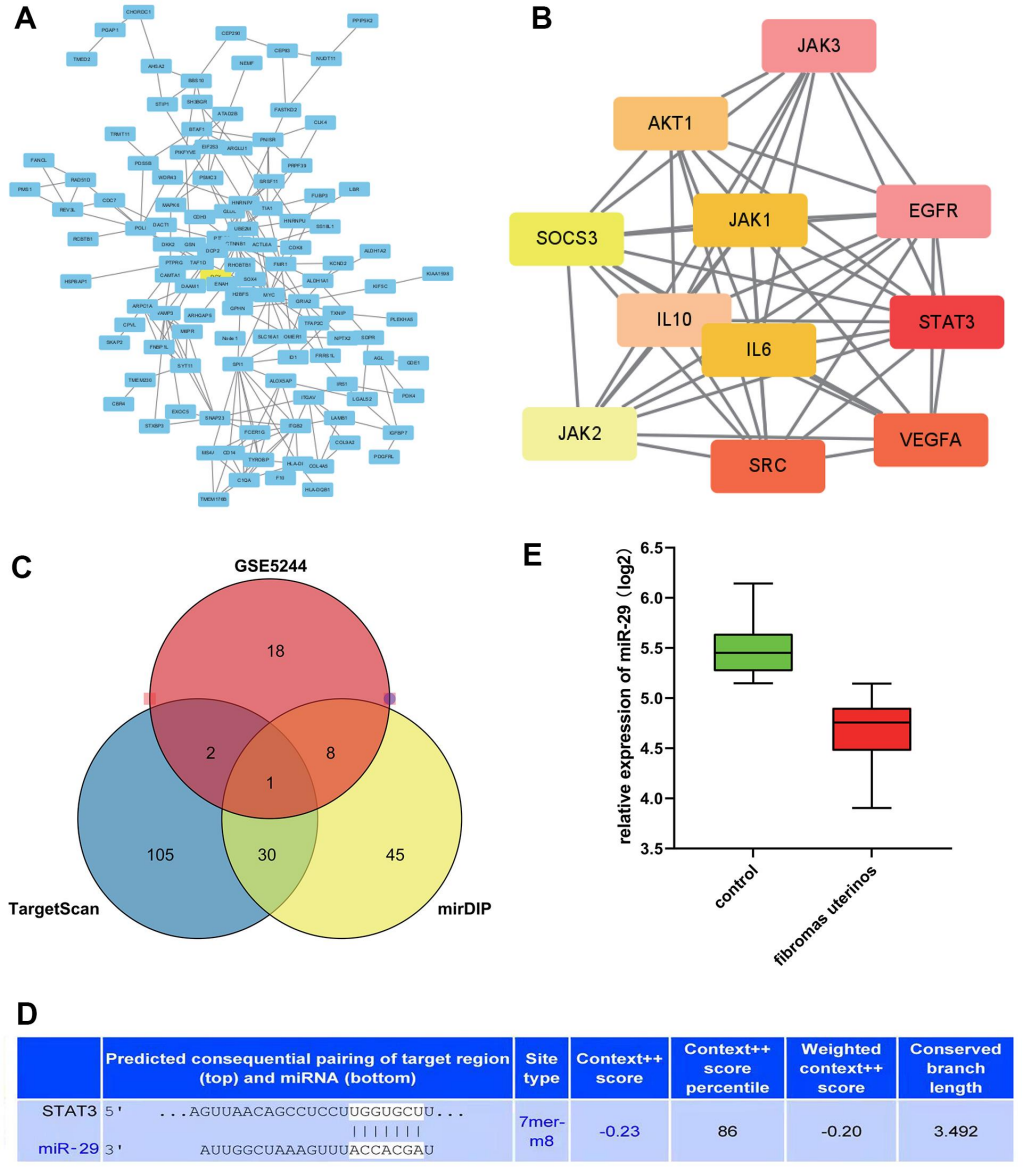
**STAT3 as the hub genes for uterine leiomyoma, and the association between miR-29 and STAT3.** (**A**) Protein-protein interaction (PPI) network. (**B**) STAT3 was in the powerful module through the Molecular Complex Detection tool (MCODE) analysis. (**C**) A Venn diagram was drawn to identify the common miRNA (miR-29c-3p) among the TargetScan, miRDB, and GSE5244. (**D**) predicted consequential pairing of the target region of STAT3 and miR-29C-3p. (**E**) compared with the control group, the expression of miR-29c was lower in the uterine leiomyoma.

Furthermore, the study conducted a protein-protein interaction (PPI) network analysis, which was performed on DEGs related to the GO pathway. The PPI network was established through the Cytoscape tool (Figure 3A) based on the STRING database. Using MCODE, we found that STAT3, VEGFA, CCND1, and other genes were the hub genes of uterine leiomyoma, proving that they might play a significant role in the pathogenesis and progression uterine leiomyoma (Figure 3B).

### The prediction between miRNA and target gene

TargetScan and miRDB online tools were applied to predict the miRNA for the target gene. Furthermore, a Venn diagram was drawn to identify the common miRNA (miR-29c-3p) among the TargetScan, mid-B, and GSE5244 (Figure 3C), which suggested that miR-29C-3p was associated with STAT3. In addition, predicted consequential pairing of a target region of STAT3 and miR-29C-3p was manifested in Figure 3D.

### Statistical analysis of target genes

**Figure 4:**
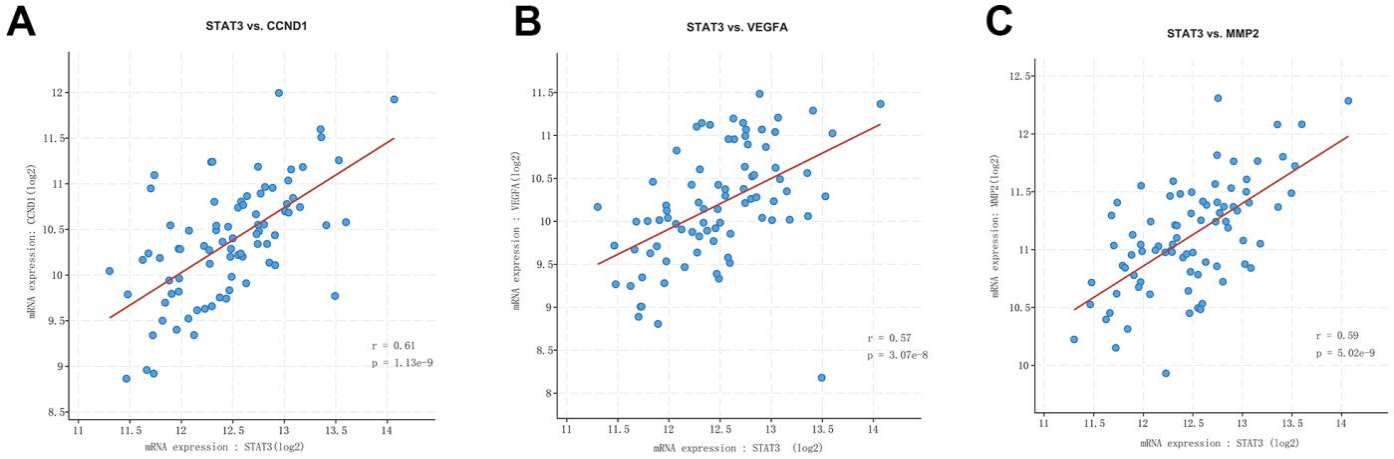
**Correlation analysis among STAT3, VEGFA, CCND1, and MMP2/9.** STAT3 was positively correlated with CCND1 (**A**), VEGFA (**B**), and MMP2/9 (**C**).

The expression level of miR-29c in different groups was analyzed. It could be seen that the expression of miR-29c was lower in the uterine leiomyoma group than in the control group (Figure 3E). Correlation analysis showed that STAT3 was positively correlated with CCND1, VEGFA, and MMP2/9, respectively (Figure 4).

### Inhibiting effect of miR-29 on HTP6136 cell migration in uterine leiomyoma cells (UtLMCs)

**Figure 5:**
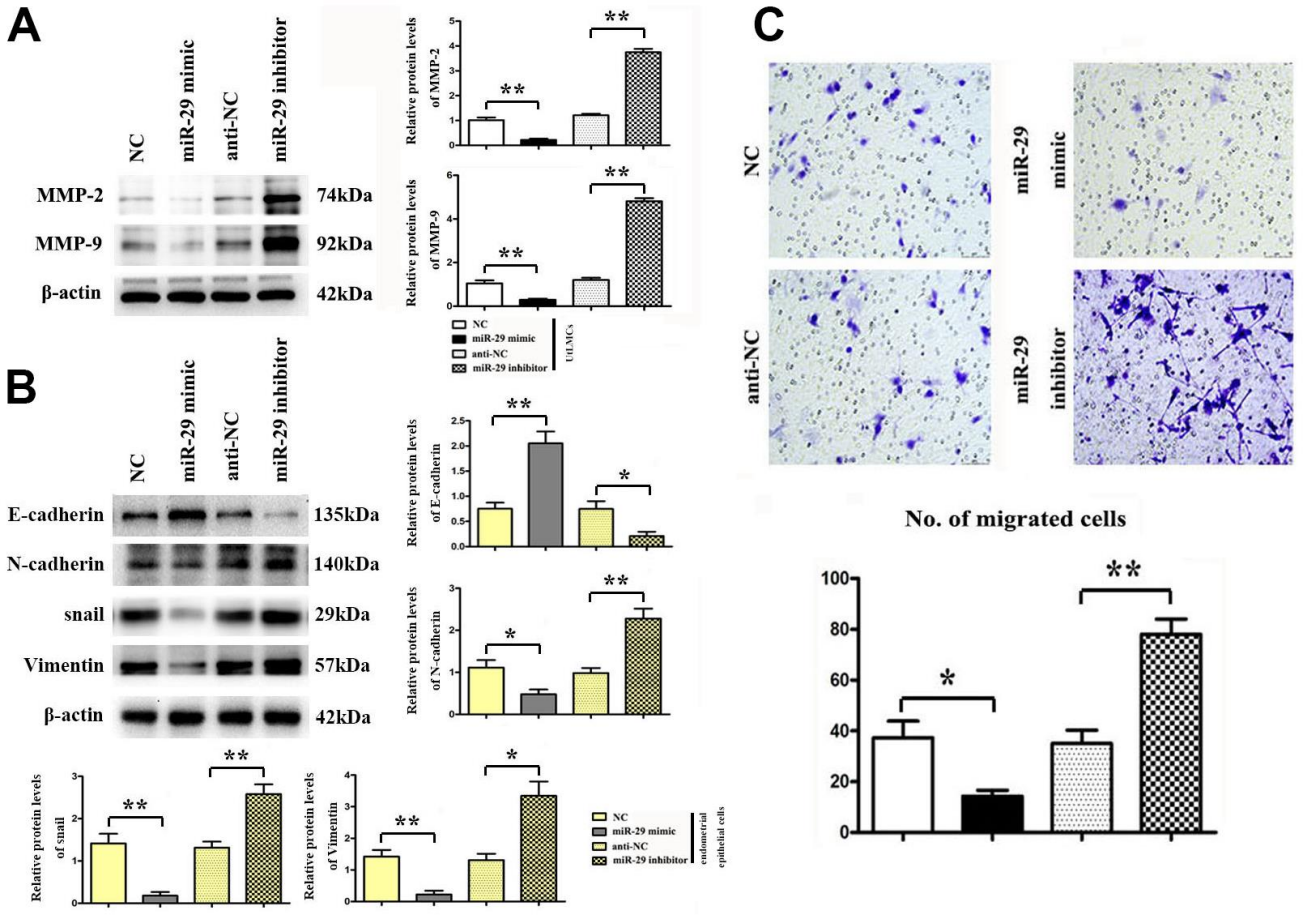
**Inhibiting effect of miR-29 on cell migration in UtLMCs and EMT in endometrial epithelial cells.** (**A**) miR-29 inhibited MMP-2 and MMP-9 *in vitro* compared with the NC control, and miR-29 inhibitors promoted the protein expression of MMP-2 and MMP-9 *in vitro* compared with the anti-NC control. (**B**) miR-29 increased the E-cadherin and suppressed the N-cadherin, snail, vimentin expression vs. NC control, and miR-29 inhibitors decreased E-cadherin and increased N-cadherin, snail, vimentin expression in endometrial epithelial cells tested by western blot. (**C**) miR-29 suppressed cell migration in UtLMCs *in vitro* compared with the NC control, and MiR-29 inhibitors promoted cell migration in UtLMCs *in vitro* compared with the anti-NC control. Data are presented as mean ± SE (*P < 0.05, **P < 0.01). UtLMCs: Uterine leiomyoma cells; NC: negative control; MMP: matrix metalloproteinases.

The expression of MMP-2 and MMP-9 proteins in UtLMCs was confirmed by western blot. β-actin was used as a loading control. Results showed that miR-29 inhibited MMP-2 and MPP-9 *in vitro* compared with the NC control, and miR-29 inhibitors promoted the protein expression of MMP-2 and MMP-9 *in vitro* compared with the anti-NC control (*P* < 0.01, Figure 5A). Transwell migration assay showed that miR-29 suppressed cell migration in UtLMCs *in vitro* compared with the NC control (*P* < 0.05), and miR-29 inhibitors promoted cell migration in UtLMCs *in vitro* compared with the anti-NC control (*P* < 0.01, Figure 5B).

### Effect of miR-29 on cell proliferation in HTP6136 uterine leiomyoma cells (UtLMCs)

**Figure 6:**
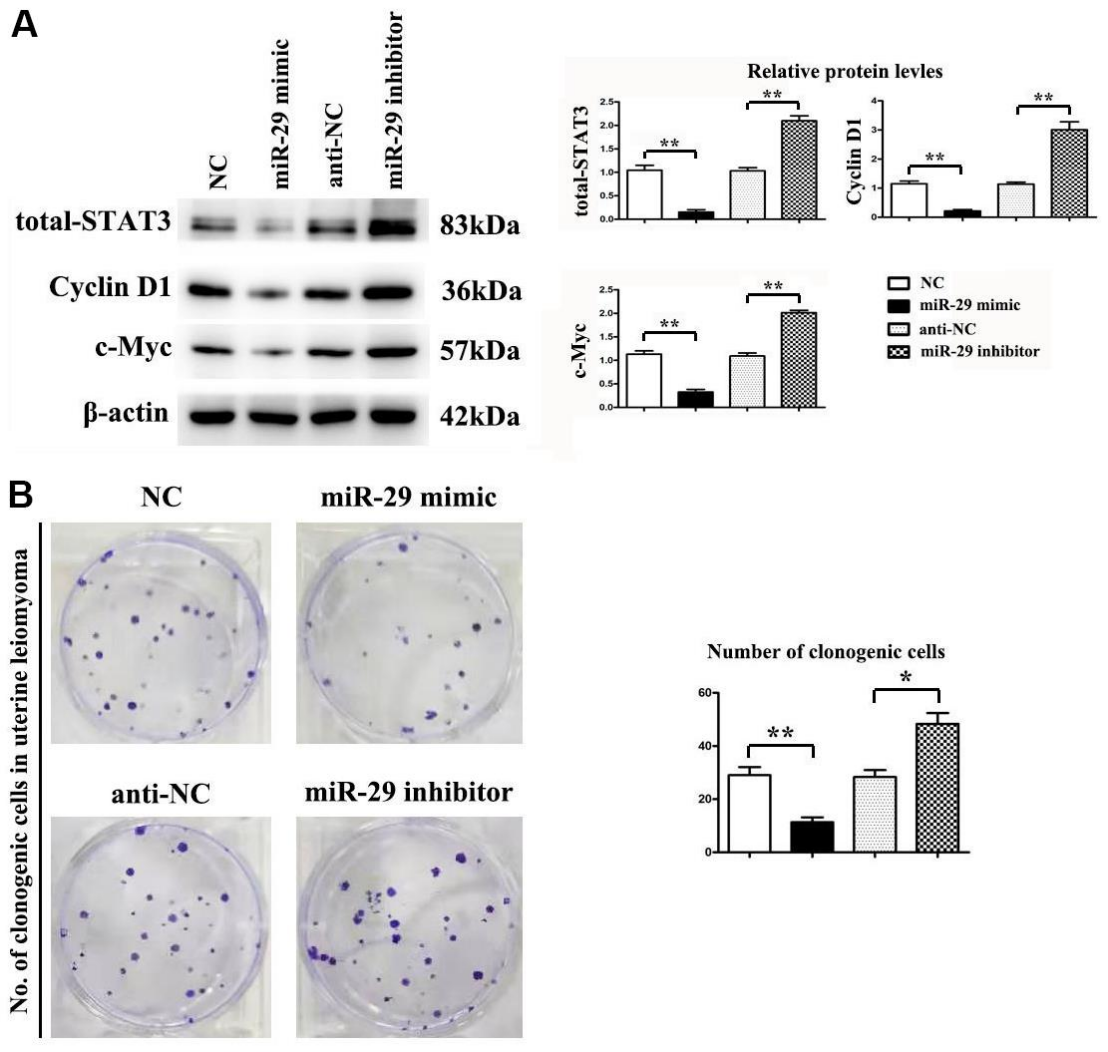
**miR-29 inhibited proliferation in uterine leiomyoma cells (UtLMCs).** (**A**) miR-29 mimics inhibited STAT-3, Cyclin D1, and c-Myc *in vitro* compared with the NC control, and MiR-29 inhibitors promoted the protein expression of STAT-3, Cyclin D1, and c-Myc *in vitro* compared with the anti-NC control. (**B**) miR-29 mimicking suppressed cell proliferation in UtLMCs *in vitro* compared with the NC control, and MiR-29 inhibitors promoted cell proliferation in UtLMCs *in vitro* compared with the anti-NC control. Data are presented as mean ± SE (*P < 0.05, **P < 0.01). UtLMCs: uterine leiomyoma cells; NC: negative control; STAT-3: signal transducer and activator of transcription 3.

Whether miR-29 mimic affected the protein expression of STAT-3, Cyclin D1, and c-Myc was assessed by western blot. β-actin was used as a loading control. Results indicated that miR-29 mimics suppressed STAT-3, Cyclin D1, and c-Myc protein expression *in vitro* compared to the NC control, while miR-29 inhibitors boosted STAT-3, Cyclin D1, and c-Myc protein expression *in vitro* compared to the anti-NC control (P < 0.01, Figure 6A). Results demonstrated that miR-29 mimics inhibited cell proliferation in UtLMCs *in vitro* compared to the NC control (P < 0.01), while miR-29 inhibitors enhanced cell proliferation in UtLMCs *in vitro* compared to the anti-NC control (P < 0.05). (Figure 6B).

## DISCUSSION

Recently, the incidence of uterine leiomyoma has been rising gradually. Uterine leiomyoma could cause urgent urination, constipation, infertility, and other reactions, resulting in a threat to the quality of life [15]. To better understand and treatment for uterine leiomyoma, the mechanisms of this disease should be carried out. Therefore, in-depth exploration of the molecular mechanism of uterine leiomyoma is significant for the research of targeted drugs [16]. The main results of this study were that the expression of miR-29 was lower in uterine leiomyoma, and the expression of STAT3 was up-regulated in uterine leiomyoma. When miR-29 was not inhibited, it could bound to STAT3 and led to the degradation of STAT3, thereby reducing the expression of Cyclin D1 and c-Myc and inhibiting the proliferation of uterine leiomyoma cells. Moreover, the expression of MMP2/9 was decreased, and the invasiveness of uterine leiomyoma cells was weakened. In contrast, when miR-29 was inhibited, the opposite result occurred.

Uterine leiomyoma is rich in the extracellular matrix. The proliferation and growth of tumor cells cannot be carried out rapidly by themselves. The proliferation and growth of uterine leiomyoma are mainly caused by the excessive production of extracellular matrix [17]. Overexpression of collagen subtypes and proteoglycans stimulates the formation of fibrotic cytokines, such as transforming growth factors, which may diminish or eliminate Matrix Metalloproteinases (MMPs) [18]. The research showed that the hyaluronic acid expression was low in normal myometrium and fibroid tissue. At the same time, myometrium and fibroid tissue had multiple levels of sulfated glycosaminoglycans like heparin sulfate, keratin in sulfate, chondroitin sulfate, and heparin. Sulfated glycosaminoglycans have the ability to release cytokines that stimulate tumor development and collagen formation. These factors all play a role in the occurrence and development of fibroids to varying degrees [19]. The expression of MMP2/9 has increased to damage the extracellular matrix, and uterine leiomyoma's invasiveness was enhanced. EMT plays a crucial role in uterine leiomyoma’ invasion and metastasis [20]. E-cadherin is one of the calcium adhesion protein family members. Its loss or reduction of expression can promote cell migration and invasion. E-cadherin is a marker molecule of phenotypic epithelial cells, which is of great value for maintaining cell polarity [21]; N-cadherin and vimentin are marker molecules of interstitial phenotypic cells, which can promote cell movement and invasion; Snail is a transcription factor that regulates the EMT process [22], which bind to the E-cadherin promoter region and hinder gene transcription, weaken the epithelial phenotype and promote its transformation to interstitial phenotype. Thereby the inhibition EMT plays a protective role in the invasion of uterine leiomyoma.

Signal transducer and activator of transcription 3 (STAT3) was a signaling protein involving various growth factors and cytokines. It mainly exists in the cytoplasm and induces transcription through phosphorylation, which plays an essential role in cell signaling [23]. STAT3 is required for cell transformation mediated by the Src oncogene, directly linked to human cancers [24]. The active STAT3 signaling pathway is a crucial transcription factor that can directly activate c-Myc, VEGF, MMP2/9, and other molecules, promoting tumor cell proliferation, invasion, metastasis, and angiogenesis [25]. One research showed that STAT3 could increase the expression of Cyclin D1 and c-Myc, and promote the proliferation of uterine leiomyoma cells [26]. In addition, the STAT3 signaling pathway enhances cell invasion by increasing the expression of MMP2 and MMP9. Furthermore, MMP9 also activates STAT3 binding sites and has a high affinity. At the same time, the STAT3 signaling pathway can play an essential role in the occurrence and development of tumors [27].

MiRNA can degrade or inhibit mRNA translation and thus participate in the regulation of cell proliferation, apoptosis, and invasion [28, 29]. The miR-29 family is composed of miR-29a, miR-29b-1, miR-29b-2, and miR-29c and is encoded by two gene families [30]. It has been reported that miR-29a can regulate the expression of the BMI1 gene through NF-κB and Wnt/β-catenin signaling pathways, thereby inhibiting the growth, migration, and invasion function of tumor cells [31]. Qiang found that miR-29b was inhibited in uterine leiomyoma, and the restoration of miR-29b expression in fibroid smooth muscle cells inhibited extracellular matrix accumulation and cell proliferation rate [32]. Chuang’s research manifested that the expression of miR-29c in uterine leiomyoma was inhibited, and its expression was negatively correlated with the expression of COL3A1 and DNMT3A. The inhibition of miR-29c expression in uterine leiomyoma is mediated by the transcriptional regulation of NF-kB and SP1 [33]. MiR-29 can also act as a tumor suppressor to inhibit the proliferation of gastric cancer, lung cancer, liver cancer, and colon cancer [34]. It has also been reported that miR-29c can inhibit the metastasis of colon cancer [35]. Arechiga-Ocampo E found that miR-29c was significantly down-regulated in model cell lines, and high expression of miR-29c reduced the radiation resistance of cells [36]. It has been suggested that the differential expression of miR-29c can be used as a potential marker for tumor diagnosis [37]. In addition, some studies have suggested that miR-29c may be a target for tumor therapy [38]. The miR-29 family is down-regulated in head and neck squamous cell carcinoma and to perform a tumor-suppressive effect by blocking the oncogene signaling pathway mediated by integrin 1 [39]. MA showed that miR-29a could target CDK6 and down-regulate JNK and p38/MAPK/ERK signaling pathways, thereby inhibiting the proliferation of schwannoma [40]. Shu YJ found that miR-29C-5p directly acted on CPEB4 and inhibited the MAPK signaling pathway to inhibit the proliferation and metastasis of bladder cancer [41]. MiR-29B-3p regulates STAT3 in lung adenocarcinoma cells, according to Liu's research, and STAT3 can boost lung adenocarcinoma cell proliferation while inhibiting apoptosis [41]. The above literature review supports the results of this experiment, so it is speculated that miR-29 may play an essential role in the growth and development of uterine leiomyoma. When miR-29 was overexpressed, it bound to STAT3, resulting in the degradation of STAT3. And then, the expression of Cyclin D1 and c-Myc was decreased, and the proliferation of uterine leiomyoma cells was inhibited. In addition, the expression of MMP2/9 was decreased, and the metastasis and invasion of uterine leiomyoma were weakened.

Mir-29 is down-regulated in tumor cells, with many ectopic productions of mir-29, which reduces the expression of stem cell-related transcription factors and their capacity to form pellets, activates the STAT3 signaling pathway, raises STAT3 expression, and promotes tumor pathogenesis. An increased mir-29 expression can inhibit tumor cell proliferation by decreasing STAT3 expression PMID: 31539114. Previous studies have shown that PLK1 and STAT3 are mutually activated and regulate the same c-Myc pool downstream. Tumor cells treated with STAT3 inhibitors exhibit lower c-Myc expression *in vitro*, co-induce cell invasion, and apoptosis, and inhibit tumor in xenografts. STAT3 can directly regulate the expression of c-Myc and induce tumor cell apoptosis by regulating c-Myc. PMID: 32962858MMPs play a key role in extracellular matrix degradation, enhancing tumor invasion, proliferation, and metastasis potential. 22349830 Studies showed that STAT3 was identified as an essential mediator of MMP2 and MMP9 expression PMID: 30410547, and STAT3 directly activated the transcription of MMP2 and MMP9 in cancer cells PMID: 25407307 PMID: 27465831. When STAT3 is missing, MMPs decreases PMID: 30040915 31583048 Mir-29 binds to STAT3 and induces degradation of STAT3. STAT3 directly affects the expression of c-Myc and MMPs, so mir-29 indirectly affects the expression of c-Myc and MMPs.

Despite the rigorous bioinformatics analysis in this paper, there are still some deficiencies. Although miR-29 is a class of small RNA consisting of only about 22 nucleotides, its function *in vivo* is very complex. MiR-29 can regulate the expression of various genes and affect the normal physiological process and the occurrence and development of diseases. In this study, no animal experiments on gene overexpression or knockout were conducted to verify its function further. Therefore, in future research, we should explore this aspect in-depth.

In conclusion, the high expression of miR-29 can inhibit cell proliferation, invasion, and metastasis in uterine leiomyoma, which might be related to the inhibition of the STAT3 signaling pathway. It can provide a novel target for diagnosing and treating uterine leiomyoma.
